# The Role of Ventricular Arrhythmias Inducibility in Arrhythmic Risk Stratification in Arrhythmogenic Right Ventricular Cardiomyopathy: A Meta‐Analysis of Observational Studies

**DOI:** 10.1002/joa3.70204

**Published:** 2025-11-01

**Authors:** George Bazoukis, Athanasios Saplaouras, Nikhil Pillai, Polyxeni Efthymiou, Sharen Lee, Dimitrios Sfairopoulos, Panagiotis Korantzopoulos, Gary Tse, Tong Liu, Konstantinos P. Letsas

**Affiliations:** ^1^ Department of Cardiology Larnaca General Hospital, State Health Services Organization Larnaca Cyprus; ^2^ Department of Basic and Clinical Sciences University of Nicosia Medical School Nicosia Cyprus; ^3^ Department of Cardiology Onassis Cardiac Surgery Center Athens Greece; ^4^ Department of Cardiology Nicosia General Hospital, State Health Services Organization Nicosia Cyprus; ^5^ Cardiac Electrophysiology Unit Cardiovascular Analytics Group Hong Kong China; ^6^ Department of Cardiology University of Ioannina, Medical School Ioannina Greece; ^7^ Tianjin Key Laboratory of Ionic‐Molecular Function of Cardiovascular Disease, Department of Cardiology Tianjin Institute of Cardiology, Second Hospital of Tianjin Medical University Tianjin China; ^8^ Kent and Medway Medical School University of Kent and Canterbury Christ Church University Canterbury Kent UK; ^9^ School of Nursing and Health Studies Hong Kong Metropolitan University Hong Kong China

**Keywords:** arrhythmogenic cardiomyopathy, ARVC, risk stratification, ventricular arrhythmias

## Abstract

**Background:**

The identification of arrhythmogenic right ventricular cardiomyopathy (ARVC) patients at risk for fatal arrhythmic events is of great importance in clinical practice. Conflicting data exist regarding the role of electrophysiological study (EPS) in this setting. We aimed to examine the association of EPS inducibility with future fatal arrhythmic events in ARVC patients and its role as a risk stratification tool in the setting of primary prevention of sudden cardiac death (SCD).

**Methods:**

This meta‐analysis was prepared in adherence to the Preferred Reporting Items for Systematic Review and Meta‐Analyses (PRISMA) guidelines.

**Results:**

Eleven studies provided data on the association between EPS inducibility and fatal arrhythmic events and were therefore included in the quantitative analysis. EPS inducibility was significantly associated with arrhythmic events (RR: 2.15, 95% CI [1.63–2.85], *p* < 0.001) in the analysis that included patients with or without prior arrhythmic events. By including only patients without prior arrhythmic events, EPS inducibility was also significantly associated with arrhythmic events (RR: 1.93, 95% CI [1.20–3.10], *p* = 0.007).

**Conclusions:**

EPS inducibility can be a valuable tool for arrhythmic risk stratification purposes in ARVC patients, especially as a component of multiparametric risk scores. More studies are needed to examine the role of multiparametric risk scores, including EPS inducibility, in identifying ARVC patients at risk for fatal arrhythmic events.

## Graphical abstract

EPS inducibility can be a valuable tool for arrhythmic risk stratification purposes in ARVC patients. It can be used as a component of multiparametric risk scores for risk stratification purposes instead of a stand‐alone decision‐making tool.

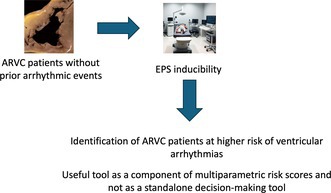

## 1 Introduction

Arrhythmogenic right ventricular cardiomyopathy (ARVC) is a group of conditions characterized by right ventricular fibrofatty infiltration, while there is a predominant arrhythmic presentation [1, 2]. Although there is clear evidence for implantable cardioverter defibrillator (ICD) implantation in patients who have suffered an aborted cardiac arrest, sustained ventricular tachycardia (VT), or ventricular fibrillation (VF), no clear criteria exist for the appropriate selection of patients in the setting of primary prevention of sudden cardiac death (SCD) [3]. In this setting, different factors have been established for risk stratification of ARVC patients. These factors include male sex, cardiogenic syncope, extent of T‐wave inversions, premature ventricular complexes count, and reduced systolic function of the right and/or left ventricle [4, 5]. Few data exist regarding the role of electrophysiological study (EPS) inducibility in the risk stratification of primary prevention ARVC patients. Current ESC guidelines recommend EPS in patients with symptoms highly suspicious for ventricular arrhythmias (Class IIb, LOE C), but not in asymptomatic individuals [6]. This study aims to examine the association of electrophysiological study inducibility with future fatal arrhythmic events in ARVC patients and to examine its role as a risk stratification tool in the setting of primary prevention of SCD.

## 2 Methods

This meta‐analysis was prepared in adherence with Preferred Reporting Items for Systematic Review and Meta‐Analyses (PRISMA) guidelines [7].

### 2.1 Aims

The aim was to evaluate the association between EPS inducibility and future fatal arrhythmic events (SCD, appropriate ICD therapies, sustained VT or VF) in ARVC patients. A subgroup analysis, including only patients without a history of aborted SCD, sustained VT or VF, was also performed to examine the role of EPS as a risk stratification tool in the primary prevention of SCD.

### 2.2 Eligibility Criteria

Studies that provided data regarding the association of EPS inducibility with fatal arrhythmic events in ARVC patients were included in our meta‐analysis. In the case of duplicate cohorts, we included the study with the greater sample size and/or the longer follow‐up. Studies that did not provide data for the studied outcome were excluded.

### 2.3 Search Strategy

Two independent investigators performed a systematic search in MedLine, EMBASE, and Cochrane databases through January 2025. The reference lists of relevant studies as well as review studies and meta‐analyses were manually screened. The following keywords were used to retrieve all relevant studies: “arrhythmogenic cardiomyopathy”, “ARVC,” “electrophysiological study,” and “inducibility”. Titles and abstracts of each study were initially screened, and those deemed potentially relevant underwent full text review. Disagreements were resolved by a third investigator.

### 2.4 Data Extraction

The following data were extracted: First author, Journal of publication, Year of publication, enrollment period, follow‐up duration, number of patients, gender, age, ARVC criteria, symptoms of patients, family history of ARVC, patients with ICD, patients with EPS inducibility, and patients with ventricular arrhythmias during follow‐up.

### 2.5 Quality Assessment

The Newcastle–Ottawa Quality Assessment Scale (NOS) was used for the quality assessment of the included studies [8]. The NOS point score system evaluated the categories of study participant selection, comparability of the results, and quality of the outcomes. This scale ranged from zero to nine stars, which indicated that studies were graded as poor quality if they scored less than 5, fair quality if they scored from 5 to 7, and good quality if they scored more than 8.

### 2.6 Statistical Analysis

Data analysis was performed by using the Review Manager software (RevMan), version 5.3 (available from the website of the Cochrane Collaboration). We performed a quantitative synthesis regarding the association between EPS inducibility and fatal arrhythmic events. The proportion of heterogeneity across studies not explained by chance was assessed by the *I* ^2^ index. A random effects model was used for the analyses. A sensitivity analysis was performed by estimating the pooled effect size after removing each study one by one. Funnel plots were constructed using RevMan software to assess publication bias. A *p*‐value of less than 0.05 (two‐tailed) was considered statistically significant.

## 3 Results

### 3.1 Study Search

**FIGURE 1:**
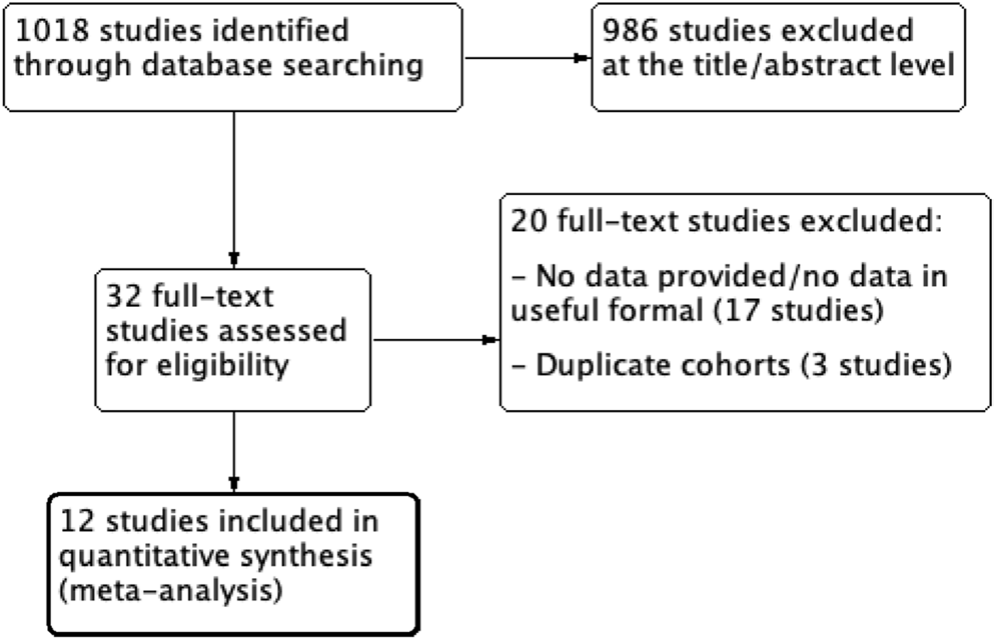
Flow diagram of the included studies.

Of the 1018 studies, 986 studies were excluded at the title/abstract level and 20 studies were excluded at the full‐text level. Finally, 12 studies [9, 10, 11, 12, 13, 14, 15, 16, 17, 18, 19, 20] provided data regarding the association between EPS inducibility and fatal arrhythmic events and were therefore included in the quantitative analysis (Figure 1).

### 3.2 Study Characteristics

The baseline characteristics and the main findings of the studies included are presented in Table 1. The population consisted of 1182 patients. In the quality assessment, all included studies were graded with a score greater than 5, and none of the included studies was characterized as having poor quality (Table S1).

**TABLE 1:** Baseline characteristics and type of arrhythmic events reported during follow‐up.

	Sample size (*N*)	Mean age	Males	Family history of ARVC	History of syncope	ICD implantation	EPS protocol	Inducibility criteria	Experienced NSVT/SVT	Diagnostic criteria of ARVC	Arrhythmic events reported during follow up
Battipaglia I, 2012	30	45.4 ± 18	17	ARVC (8/30)	9	15	Not available	Sustained VT and/or tachyarrhythmias with rapid hemodynamic compromise requiring immediate defibrillation was induced	Not reported	1994	VT, VF
Chung FP, 2016	63	44.7 ± 14.8	38	SCD (10/63)	28	Not reported	PVS protocol: up to three extrastimuli delivered during sinus rhythm and after eight paced ventricular cycle lengths Pacing: right ventricular apex, followed by pacing from the right ventricular outflow tract in cases without inducible sustained ventricular arrhythmias from the right ventricular apex Isoproterenol (1–4 μg/min) was administered intravenously to facilitate induction of ventricular arrhythmia if no tachycardia was induced during baseline programmed electrical stimulation	Not available	Not reported	2010	SCD, VA
Corrado D, 2010	106	35.6 ± 18	71	SCD (49/106)	42	106	A minimum of 2 drive‐cycle lengths and up to 3 ventricular extrastimuli were used while pacing from 2 right ventricular sites	Sustained ventricular tachyarrhythmia (VT, VF, or ventricular flutter) that lasted > 30 s or required termination because of hemodynamic compromise	0	1994	Appropriate ICD therapy
Gasperetti A, 2022	288	41.0 ± 14.5	161	ARVC (76/288)	69	78	A 3 extra stimuli PVS protocol was used, delivered at 2 right ventricle sites (right ventricular apex and outflow tract)	Induction of a sustained monomorphic VT lasting ≥ 30 s or leading to hemo‐dynamic compromise. Induction of polymorphic VT and VF/ventricular flutter was not considered a positive PVS	134	2010	Appropriate ICD intervention, SCD, SVT
Heidbuchel H, 2003	46	31 (16–53)	45	Not reported	30	0	Fixed rate pacing (at cycle lengths down to 240 ms) and delivery of up to three extrastimuli at basic cycle lengths of 600 and 400 ms, both in the right ventricular apex and outflow tract (down to the ventricular refractory period but never < 180 ms) In all but three athletes a similar stimulation protocol was applied during isoproterenol infusion (1–4 μg/min) if the baseline study was negative	Induction of sustained VT or VF	9	1994	SVT, SCD
Maupain C, 2018	137	37 ± 13	107	SCD (24/137)	19	0	PVS: 2 sites (right ventricular apex and RV outflow tract) at twice pacing threshold and a 2‐ms duration PVS was first performed without isoproterenol at 600‐ and 400‐ms basal cycle length (8 paced S1 beats) and up to 3 extrastimuli with a 10‐ms decrement interval from 350 ms until the ventricular refractory period or a minimum 200‐ms coupling interval If no sustained VT or ventricular fibrillation was induced as baseline, PVS was performed under isoproterenol (1–4 mg/min) infusion (target heart rhythm of 120 beats/min or 50% increase of basal cardiac frequency), and PVS was performed at 2 sites (RV apex and RV outflow tract) at a 400‐ms basal cycle length (8 S1 beats) with up to 3 extrastimuli with a 10‐ms decrement from 350 ms to the ventricular refractory period or a minimum 200‐ms coupling interval	Induction of sustained VT or VF	94	2010	VA
Migliore F, 2013	69	35 (28–45)	47	ARVC/D (28/69)	Cardiac arrest/syncope 22	31	PVS protocol included 3 drive cycle lengths (600, 500, and 400 ms) and 3 ventricular extrastimuli while pacing from 2 right ventricular sites (apex and outflow tract) PVS was repeated after intravenous isoproterenol infusion in those patients with effort induced non sustained VT	VF or sustained VT (i.e., one that lasted ≥ 30 s or required termination because of hemodynamic compromise)	81	1994	VT, VF, SCD
Orgeron GM, 2017	312	33.6 ± 13.9	163	ARVC (60/312)	96	312	Not available	Sustained VT—VT or VF that lasted ≥ 30 s or that required termination due to hemodynamic compromise	114	2010	VT, VF
Pezawas T, 2006	34	49 ± 12	21	ARVC/SCD (14/34)	17	16	Three extrastimuli delivered during sinus rhythm and after eight paced ventricular cycle lengths at 500, 430, 375, and 333 ms. First the right ventricular apex, then the right ventricular outflow tract was tested in case no sustained ventricular arrhythmia was induced previously	Induction of a sustained monomorphic VT	29	1994	VT
Saguner AM, 2013	62	42.3 ± 13.5	42	0	31	Not reported	Delivery of up to three extrastimuli at three basic cycle lengths and ventricular burst pacing at two right ventricular sites (right ventricular apex and outflow tract) In cases of no inducibility isoproterenol was administrated intravenously (4 mg/min), followed by application of 3 extra stimuli and burst pacing (to a minimum of 250 ms)	Monomorphic VT was sustained or required termination because of hemodynamic compromise The presence of inducible VF or polymorphic VT was not considered a positive EPS	14	2010	VT, VF, SCD
Xue SL, 2019	35	38.6 ± 11.0	15	Not reported	3	3	Intravenous isoproterenol (1–4 mg/min) was administered if sustained VT was not induced by the baseline EPS PVS consisting of up to three extra stimuli was performed at the right ventricular apex and right out ow tract by delivering current at twice the diastolic threshold. Coupling intervals were progressively shortened until a response was no longer elicited or to a minimum of 200 ms	Sustained VT or VF	28	1994	SCD, VA
Bhonsale A, 2011	84	31.9 ± 11.9	39	30	23	84	Not available	Sustained ventricular tachyarrhythmia—VT or VF that lasted 30 s or required termination because of hemodynamic compromise	41	2010	SVA

Abbreviations: ARVC, arrhythmogenic right ventricular cardiomyopathy; ICD, implantable cardioverter defibrillator; NSVT, non‐sustained ventricular tachycardia; PVS, programmed ventricular stimulation; SCD, sudden cardiac death; SVA, sustained ventricular arrhythmia; SVT, sustained ventricular tachycardia; VA, ventricular arrhythmias; VF, ventricular fibrillation; VT, ventricular tachycardia.

### 3.3 Main Results

#### 3.3.1 Association of EPS Inducibility and Fatal Arrhythmic Events in ARVC Patients (With or Without Prior Fatal Arrhythmic Events)

**FIGURE 2:**
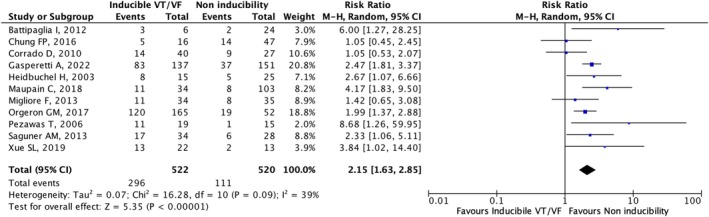
Forest plot of the association of EPS inducibility with arrhythmic events in ARVC patients (with or without prior fatal arrhythmic events).

The analysis included 11 studies that provided data about the association between EPS inducibility and fatal arrhythmic events in ARVC patients (with or without prior events). The population comprised 1042 patients. The quantitative synthesis showed that EPS inducibility was significantly associated with fatal arrhythmic events (RR: 2.15, 95% CI [1.63–2.85], *p* < 0.001) (Figure 2). Visual inspection of the funnel plot asymmetry did not reveal significant publication bias. No significant heterogeneity among the included studies (*I* ^2^ = 39%; *p* = 0.09) was observed. Sensitivity analysis demonstrated that, when including only studies using the 2010 diagnostic criteria, the association between EPS inducibility and fatal arrhythmic events remained significant (RR: 2.22, 95% CI [1.66–2.99], *p* < 0.001). Similarly, the association remained significant when including studies using the 1994 diagnostic criteria [RR: 2.30, 95% CI (1.25–4.24), *p* < 0.01]. Furthermore, excluding studies that included patients who underwent catheter ablation procedures, the association between EPS inducibility and future fatal arrhythmic events remained significant (RR: 1.88, 95% CI [1.07–3.30], *p* = 0.03).

#### 3.3.2 Association of EPS Inducibility With Arrhythmic Events in ARVC Patients Without a History of Fatal Arrhythmic Events

**FIGURE 3:**
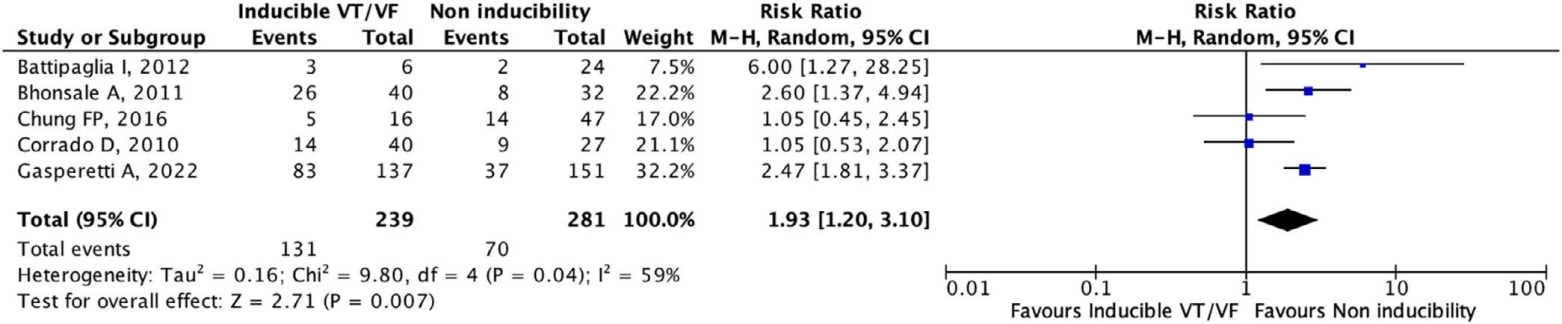
Forest plot of the association of EPS inducibility with arrhythmic events in ARVC patients without a history of fatal arrhythmic events.

The analysis included five studies providing data about the association between EPS inducibility and fatal arrhythmic events in ARVC patients without a prior history of such events. The population consisted of 520 patients. The quantitative synthesis showed that EPS inducibility was significantly associated with fatal arrhythmic events (RR: 1.93, 95% CI [1.20–3.10], *p* = 0.007) (Figure 3). Visual inspection of the funnel plot asymmetry did not reveal a significant publication bias. A significant heterogeneity among the included studies (*I* ^2^ = 59%; *p* = 0.04) was observed. Sensitivity analysis showed that including only studies that used the 2010 diagnostic criteria maintained a significant association between EPS inducibility and fatal arrhythmic events (RR: 2.12, 95% CI [1.37–3.29], *p* < 0.001), while no significant association was found in the quantitative synthesis of the two studies that used the 1994 diagnostic criteria (RR: 2.17, 95% CI [0.40–11.70], *p* = 0.37). Furthermore, excluding Chung et al. [11], which included patients who underwent catheter ablation, the association between EPS inducibility and future fatal arrhythmic events remained significant (RR: 2.18, 95% CI [1.33–3.57], *p* = 0.002).

## 4 Discussion

This study showed a significant association between EPS inducibility and future fatal arrhythmic events in the analysis that included patients with and without prior fatal arrhythmic events. Similarly, a significant association was also found in the analysis that included only patients without a history of prior fatal arrhythmic events.

The arrhythmic risk stratification of ARVC patients, especially for those patients with no prior fatal arrhythmic event, is the cornerstone for the selection of patients who need ICD implantation. Different factors have been examined for their prognostic role in predicting future arrhythmic events in ARVC patients. Results from a meta‐analysis showed that male sex, syncope, T‐wave inversion in lead > V3, right ventricular dysfunction, and prior (non)sustained VT/VF consistently predicted ventricular arrhythmias in all populations with ARVC [21]. Another meta‐analysis showed that predictors of life‐threatening arrhythmic events including sudden cardiac death were age at presentation, male sex, right ventricular (RV) dysfunction, QRS fragmentation, T‐wave inversion, syncope at presentation, previous non‐sustained ventricular tachyarrhythmia, and the task force criteria score [22]. In the same study, predictors of appropriate ICD therapy were RV dysfunction, syncope, and inducible ventricular arrhythmia [22]. Similarly, male gender, presyncope, left ventricular dysfunction, T‐wave inversions in inferior leads, proband status, late potentials, syncope, inducibility at EPS, right ventricular dysfunction, epsilon waves, and premature ventricular contractions greater than 1000/24 h were identified as predictors of arrhythmic events in another meta‐analysis [5]. Regarding electrocardiographic variables, a recent meta‐analysis showed that epsilon waves, prolonged terminal activation duration of QRS, fragmented QRS, late potentials on signal‐averaged electrocardiogram, T‐wave inversion in right precordial leads, and extension of T‐wave inversion in inferior leads have been associated with increased risk of arrhythmic events [23].

Conflicting data exist regarding the role of EPS inducibility in the risk stratification of ARVC patients. Corrado et al. included 106 ARVC patients who received an ICD based on 1 or more arrhythmic risk factors, such as syncope, nonsustained ventricular tachycardia, family history of sudden death, and inducibility at programmed ventricular stimulation [12]. In this study, the positive predictive value of programmed ventricular stimulation was 35% for any appropriate ICD intervention and 20% for shocks for VF/ventricular flutter, with a negative predictive value of 70% and 74% [12]. According to these findings, preimplantation EPS has limited value in identifying individuals at risk of a first episode of lethal ventricular arrhythmia. On the other hand, a multicenter study included 288 patients with a definite ARVC diagnosis, no history of sustained ventricular arrhythmias at diagnosis, and EPS performed at baseline [13]. In this study, inducible ventricular tachycardia predicted clinical sustained ventricular arrhythmias during the 5‐year follow‐up. Compared with the ARVC risk calculator alone, the addition of EPS inducibility showed improved prediction of ventricular arrhythmia events [13]. In a small cohort of 84 patients who underwent ICD implantation for primary prevention, inducibility at EPS and nonsustained ventricular tachycardia were independent strong predictors of appropriate ICD therapy [10]. Male sex, presence of a pathogenic mutation, inducibility at EPS, major RV structural disease, and lack of family history of ARVC have been associated with an increased risk of arrhythmic outcome in late presentation ARVC patients [24].

Different factors can influence the prognostic role of EPS in ARVC patients. In a cohort of ARVC patients without ICD, EPS was effective in identifying a substrate for developing re‐entrant monomorphic VTs but was less effective in predicting lethal arrhythmic events [15]. Different mechanisms of SCD that are not predictable by EPS could explain its limited utility in risk stratification for primary prevention patients. These mechanisms include focal VT, PVC‐triggered VF, or SCD triggered by an acute event [15]. Interestingly, it has been proposed that during the early phase of the disease, VT is an epiphenomenon, while during the late phase of the disease, severe heart failure may be responsible for VT occurrence [25]. The diagnostic criteria used for ARVC diagnosis may also influence the prognostic role of EPS inducibility. Specifically, Piccini JP et al. showed that patients with probable ARVC and a negative EPS did not experience appropriate ICD intervention [26] The negative prognostic value of EPS in patients with probable ARVC could be useful in risk stratification. Similarly, the induction of VT during EPS has been identified as the most significant predictor for ICD firing in patients with a definitive diagnosis of ARVC [27]. In contrast, results from a meta‐analysis indicated that inducibility during EPS predicted ventricular arrhythmias only in patients with borderline and not definite ARVC [21].

## 5 Limitations

Our study has several limitations. Firstly, it included observational studies, which may introduce selection biases that could influence our results. Additionally, no data were available in the included studies regarding EPS reproducibility at the individual level, raising questions about its reliability. Differences in EPS protocols and definitions of positive programmed ventricular stimulation across the included studies could substantially affect the outcomes of this meta‐analysis, primarily by introducing heterogeneity. The EPS protocols used were not specified in two of the included studies (Table 1). Although a sensitivity analysis could not be performed based on the EPS protocol, we employed random‐effects models for the quantitative synthesis. The usage of a standardized EPS protocol in future research would enhance comparability and improve the validity of pooled estimates. Other factors that could influence the association between EPS inducibility and future arrhythmic events include the use of antiarrhythmic agents and the proportion of patients with exercise restrictions during follow‐up. Regarding diagnostic criteria, we included studies that used either the 1994 [28] or 2010 [29] diagnostic criteria. The usage of these criteria and the classification of patients as having a definite, borderline, or possible diagnosis of ARVC could affect the results. The performance of ARVC risk calculators may be influenced by the presence of likely pathogenic or pathogenic variants [30]. The presence of likely pathogenic or pathogenic variants can also impact the prognostic value of EPS inducibility. Therefore, additional studies are needed to clarify the role of EPS in specific populations.

## 6 Conclusions

EPS inducibility can be a valuable component of multiparametric risk scores for identifying patients at risk of fatal arrhythmic events and who may benefit from ICD implantation. However, additional studies are needed to evaluate the role of these combined risk scores, including EPS inducibility, in identifying ARVC patients without prior arrhythmic events who are at increased risk of potentially fatal arrhythmias.

## Ethics Statement

The authors have nothing to report.

## Consent

The authors have nothing to report.

## Conflicts of Interest

The authors declare no conflicts of interest.

## Supporting information


**Table S1:** Quality assessment of the included studies.

## Data Availability

The authors have nothing to report.
