# Supplementary material for: The Role of Ventricular Arrhythmias Inducibility in Arrhythmic Risk Stratification in Arrhythmogenic Right Ventricular Cardiomyopathy: A Meta‐Analysis of Observational Studies
Source: J Arrhythm. 2025 Nov 1;41(6):e70204. doi: 10.1002/joa3.70204 (PMC12579493; doi:10.1002/joa3.70204)
Supplement: Supplementary file 1 — Table S1: Quality assessment of the included studies. [file JOA3-41-e70204-s001.docx]

**Supplementary Table**. Quality assessment of the included studies

| First Author, Publication Year | Representativeness of the exposed cohort | Selection of the non-exposed cohort | Ascertainment of exposure | Outcome of interest not present at start of study | Comparability | Assessment of outcome | Adequacy of duration of follow-up | Adequacy of completeness of follow-up | Total score  (0-9) |
| --- | --- | --- | --- | --- | --- | --- | --- | --- | --- |
| Battipaglia, 2012^1^ | 1 | 0 | 1 | 1 | 1 | 1 | 1 | 1 | 7 |
| Bhonsale, 2011^2^ | 1 | 0 | 1 | 1 | 1 | 1 | 1 | 1 | 7 |
| Chung, 2016^3^ | 1 | 0 | 1 | 1 | 1 | 1 | 0 | 1 | 6 |
| Corrado, 2010^4^ | 1 | 0 | 1 | 1 | 1 | 1 | 1 | 1 | 7 |
| Gasperetti, 2022^5^ | 1 | 0 | 1 | 1 | 1 | 1 | 1 | 1 | 7 |
| Heidbuchel, 2006^6^ | 1 | 0 | 1 | 1 | 0 | 1 | 1 | 1 | 6 |
| Maupain, 2018^7^ | 1 | 0 | 1 | 1 | 1 | 1 | 1 | 1 | 7 |
| Migliore, 2013^8^ | 1 | 1 | 1 | 1 | 1 | 1 | 1 | 1 | 8 |
| Orgeron, 2017^9^ | 1 | 0 | 1 | 1 | 1 | 1 | 1 | 1 | 7 |
| Pezawas, 2006^10^ | 1 | 0 | 1 | 1 | 1 | 1 | 1 | 1 | 7 |
| Saguner, 2013^11^ | 1 | 1 | 1 | 1 | 1 | 1 | 1 | 1 | 8 |
| Xue, 2019^12^ | 1 | 0 | 1 | 1 | 1 | 1 | 1 | 1 | 7 |

1 Battipaglia, I, Scalone, G, Macchione, A, Pinnacchio, G, Laurito, M, Milo, M et al. Association of heart rate variability with arrhythmic events in patients with arrhythmogenic right ventricular cardiomyopathy/dysplasia. Circ J. 2012; 76: 618-623.

2 Bhonsale, A, James, CA, Tichnell, C, Murray, B, Gagarin, D, Philips, B et al. Incidence and predictors of implantable cardioverter-defibrillator therapy in patients with arrhythmogenic right ventricular dysplasia/cardiomyopathy undergoing implantable cardioverter-defibrillator implantation for primary prevention. J Am Coll Cardiol. 2011; 58: 1485-1496.

3 Chung, FP, Lin, YJ, Chong, E, Chang, SL, Lo, LW, Hu, YF et al. The Application of Ambulatory Electrocardiographically-Based T-Wave Alternans in Patients with Arrhythmogenic Right Ventricular Dysplasia/Cardiomyopathy. Can J Cardiol. 2016; 32: 1355 e1315-1355 e1322.

4 Corrado, D, Calkins, H, Link, MS, Leoni, L, Favale, S, Bevilacqua, M et al. Prophylactic implantable defibrillator in patients with arrhythmogenic right ventricular cardiomyopathy/dysplasia and no prior ventricular fibrillation or sustained ventricular tachycardia. Circulation. 2010; 122: 1144-1152.

5 Gasperetti, A, Carrick, RT, Costa, S, Compagnucci, P, Bosman, LP, Chivulescu, M et al. Programmed Ventricular Stimulation as an Additional Primary Prevention Risk Stratification Tool in Arrhythmogenic Right Ventricular Cardiomyopathy: A Multinational Study. Circulation. 2022; 146: 1434-1443.

6 Heidbuchel, H, Hoogsteen, J, Fagard, R, Vanhees, L, Ector, H, Willems, R et al. High prevalence of right ventricular involvement in endurance athletes with ventricular arrhythmias. Role of an electrophysiologic study in risk stratification. Eur Heart J. 2003; 24: 1473-1480.

7 Maupain, C, Badenco, N, Pousset, F, Waintraub, X, Duthoit, G, Chastre, T et al. Risk Stratification in Arrhythmogenic Right Ventricular Cardiomyopathy/Dysplasia Without an Implantable Cardioverter-Defibrillator. JACC Clin Electrophysiol. 2018; 4: 757-768.

8 Migliore, F, Zorzi, A, Silvano, M, Bevilacqua, M, Leoni, L, Marra, MP et al. Prognostic value of endocardial voltage mapping in patients with arrhythmogenic right ventricular cardiomyopathy/dysplasia. Circ Arrhythm Electrophysiol. 2013; 6: 167-176.

9 Orgeron, GM, James, CA, Te Riele, A, Tichnell, C, Murray, B, Bhonsale, A et al. Implantable Cardioverter-Defibrillator Therapy in Arrhythmogenic Right Ventricular Dysplasia/Cardiomyopathy: Predictors of Appropriate Therapy, Outcomes, and Complications. J Am Heart Assoc. 2017; 6

10 Pezawas, T, Stix, G, Kastner, J, Schneider, B, Wolzt, M & Schmidinger, H. Ventricular tachycardia in arrhythmogenic right ventricular dysplasia/cardiomyopathy: clinical presentation, risk stratification and results of long-term follow-up. Int J Cardiol. 2006; 107: 360-368.

11 Saguner, AM, Medeiros-Domingo, A, Schwyzer, MA, On, CJ, Haegeli, LM, Wolber, T et al. Usefulness of inducible ventricular tachycardia to predict long-term adverse outcomes in arrhythmogenic right ventricular cardiomyopathy. Am J Cardiol. 2013; 111: 250-257.

12 Xue, SL, Hou, XF, Sun, KY, Wang, Y, Qian, ZY, Wang, QP et al. Microvolt T-wave alternans complemented with electrophysiologic study for prediction of ventricular tachyarrhythmias in patients with arrhythmogenic right ventricular cardiomyopathy: a long-term follow-up study. Chin Med J (Engl). 2019; 132: 1406-1413.
